# The use of theatre in medical education in the emergency cases school: an appealing and widely accessible way of learning

**DOI:** 10.1007/s40037-017-0350-4

**Published:** 2017-04-12

**Authors:** Christodoulos Keskinis, Vasileios Bafitis, Panagiota Karailidou, Christina Pagonidou, Panteleimon Pantelidis, Alexandros Rampotas, Michail Sideris, Georgios Tsoulfas, Dimitrios Stakos

**Affiliations:** 1 Department of Medicine, Democritus University of Thrace, Alexandroupolis, Greece; 2 Department of Medicine, Aristotle University of Thessaloniki, Thessaloniki, Greece; 3 Royal London Hospital Renal Medicine and Transplantation Department, London, UK; 4 London Deanery, London, UK; 5 First Department of Surgery, Aristotle University of Thessaloniki, Thessaloniki, Greece; 6 Department of Cardiology, Democritus University of Thrace, Alexandroupolis, Greece

**Keywords:** Education, Simulation, Theatre

## Abstract

**Introduction:**

Theatre models in medical education have been used worldwide in order to train medical students and graduates in managing various situations. However, the literature reports little regarding its appeal to trainees. We conducted a medical seminar, entitled Emergency Cases School, which employed such techniques. Actors simulated the actions of doctors and patients involved in various emergency cases, in front of a large audience, in a specially modified theatre hall which resembled the emergency room environment.

**Methods:**

A total of 303 undergraduate medical students participated in the seminar. The audience evaluated the course with the DREEM questionnaire, along with two extra questions: Q1. ‘Do you think that the course will prove itself beneficial to your clinical skills?’ and Q2. ‘Would you suggest the course to another student?’, in a 0–4 scoring scale. Of the attendees, 281 (92.7%) answered the questionnaire.

**Results:**

The overall DREEM score was 140.32 (±23.39) out of 150, which is interpreted as ‘More positive than negative’. The results of Q1 and Q2 were 3.07 (±0.78) and 3.65 (±0.61), respectively.

**Discussion:**

The Emergency Cases School received positive feedback as a theatre educational tool, targeted to a large audience. With the advantage of the realistic setting of an emergency room, along with its low-budget needs, this course model could function as a creative alternative of the more traditional lecturing teaching techniques.

## Introduction

The emergency room (ER) would be the ideal ‘arena’ for training students in medical emergencies; however it is not always an accessible and appropriate place for training. The intense environment, the workload and urgency in terms of the management of critical cases makes it a far from ideal place for the simultaneous teaching of many students. Mistakes are an essential part of the learning process and in the ER there is no room for error [1–3].

Theatre techniques seem to be effective and increasingly used as interactive teaching tools in undergraduate medical education [3–12]. This simulation method of learning does not only enhance the students’ comprehension of the doctor-patient relationship, but it also improves their communicational skills and gives them the ability to engage more with the patient [4–8, 13–16]. Although theatre teaching as an educational model has numerous advantages in education, which have been described in literature, the interactive educational environment it provides from the stage, as well as its appeal to the audience, have not been evaluated [1].

After taking into consideration all these challenges of theatre-playing techniques, we decided to organize a seminar which involved such methods and applied to medical students’ need for interactive, fully accessible education. The anticipated outcome from the application of theatre-learning techniques was to train a large audience in a realistic ER-simulated environment. It is stated that the learning environment could have a significant influence on students’ future clinical performance [17]. The purpose of this project was to evaluate the learning environment created with this ‘one-to-many’ technique, as a variable that might motivate students to perceive knowledge and thus to make them achieve a better clinical performance over time. We employed the Dundee Ready Education Environment Measure (DREEM) inventory to measure the appeal of this educational environment [18]. The seminar ‘Emergency Cases School’ was held on 28 November 2015.

## Methods

### The seminar setting

The Emergency Cases School is a real-time ER-environment-simulation educational tool. Students and academic professors from two neighbouring universities (Democritus University of Thrace and Aristotle University of Thessaloniki) cooperated in order to organize and evaluate this seminar. The intervention occurred in an amphitheatre with 303 medical students. There was no admission fee and the audience consisted of students of various schools and years of study. As long as there were available seats, younger students who wanted to become familiar with the ER clinical environment were allowed to attend. This contributed to a diverse range of the years of schooling. We designed the stage of the theatre to resemble the ER of a tertiary hospital centre. The seminar included nine different problem-based case reports focused on the differential diagnosis and treatment using theatrical scenarios to present them (Table 1). The concept of each scenario was defined by the organizing committee. The organizing committee also selected carefully the instructors for each scenario with high-standard quality criteria which included the following: Firstly, we sent an initial invitation to several potential instructors. An analytic review of the course and the specific tasks for each scenario were included in the invitation. Secondly, we arranged an interview with the potential instructors which defined the final faculty of instructors. The faculty of instructors arranged the analytic script of each scenario, along with the necessary equipment and the scenery settings. After the initial design, the faculty reviewed the whole structure, optimized the details and approved the final changes. Each scenario included two parts. The 30-minute first part involved a standardized patient demonstrating a diagnosis-oriented medical history with the associated clinical symptoms. In each scenario, one or two actors along with qualified tutors portrayed the role of the doctors (consultant and resident) while a diagnostic and treatment strategy unfolded. The 15-minute second part included a scenario debrief with a qualified tutor, discussion, and additional questions and answers with the learners.

**Table 1:** The topics of the scenarios of the Emergency Cases School

Scenario	System	Differential diagnosis	Final diagnosis	Actors, including the academic tutors (total number)
1	Gastrointestinal	Peptic ulcer disease Myocardial infarction Cholecystitis Gastric cancer Oesophagitis	Acute pancreatitis	4
2	Cardiovascular	Acute aortic dissection Acute pericarditis Pulmonary embolism Infective endocarditis	Myocardial infarction	4
3	Cardiovascular	Deep venous thrombosis Vasculitis Vasospasm Peripheral vascular injury	Acute lower limb ischaemia	3
4	Nervous	Subdural/epidural/endocranial haematoma Migraine Meningitis, encephalitis Transient ischaemic attack	Subarachnoid haemorrhage	4
5	Urinary	Obstructive blood clot in the ureter Renal vein thrombosis Biliary colic Appendicitis Ureter carcinoma	Ureterolithiasis	3
6	Gastrointestinal	Acute cholangitis Acute pancreatitis Acute gastritis Inflammatory bowel disease Gastroesophageal reflux disease	Duodenal ulcer	4
7	Nervous	Seizure Systemic infection Brain tumour Metabolic disorders Vertigo	Cerebrovascular accident	4
8	Pulmonary	Acute respiratory distress syndrome Pneumonia Chronic obstructive pulmonary disease Pulmonary embolism Myocardial infarction	Under tension pneumothorax	3
9	Nervous	Multiple sclerosis Neoplasms of the spinal cord Diabetic neuropathy	Cauda equina syndrome	4

A team, consisting of these two or three trained actors, three medical students or young doctors and one academic professor as supervisor, was responsible for conducting each scenario. Apart from the supervision process, the academic teachers also had the role of the consultant doctor in each scenario, in order to control the flow of the scenario and advise the resident-actor during the history-taking, the clinical examination, the differential diagnosis and the treatment. Each scenario addressed a different medical condition with a wide differential diagnosis, so that the attendants were pushed to think of many possible diagnoses one by one, until the final condition is revealed. We used a set of special equipment for each scenario according to its needs. In order to display lab and imaging test results towards the large audience, we employed a video projector. In total, the seminar lasted nearly 7.5 h, with a 45-minute break after the first five scenarios. The total cost of the seminar was approximately €550. Specifically, €120 were spent for the sound, light and other equipment needed, €230 for the material distributed to each attendant (notebook, pen, bag, pin-tags with barcode label) and €200 for the lunch break. The university kindly offered the necessary materials and equipment for each scenario free of charge. The instructors also offered to participate for free. We obtained an ethical approval statement for the conduction and the evaluation of the seminar by the Institutional Review Board of the Democritus University of Thrace. The project was carried out in accordance with the Declaration of Helsinki, there was no potential harm to the participants, the anonymity of participants was guaranteed and the informed consent of participants was obtained.

### Evaluation

Although the idea of involving actors as standardized patients to simulate clinical signs and symptoms in front of a large audience for educational reasons has been widely used, the participants’ opinion of this particularly novel strategy of learning has not been evaluated extensively. The organizing committee of the Emergency Cases School decided to use the DREEM questionnaire as a tool to evaluate the educational environment of the seminar. Since its first development [18], the DREEM inventory has been translated and validated in many different languages [19–21]. It is one of the most widely used tools in evaluating educational environments [22–28], including the learning environment of single, brief courses and seminars as the one of the Emergency Cases Schools [29–31].

The DREEM inventory includes a set of 50 five-point Likert questions, with a score scale, from 0 to 4. For most of the questions, 4 stands for strongly agree, 3 for agree, 2 for uncertain, 1 for disagree and 0 for strongly disagree. The 50-item DREEM has a maximum score of 200 indicating the ideal educational environment, and a minimum score of 0, indicating a very worrisome result. The interpretation of the overall score is: 0–50: very poor, 51–100: plenty of problems, 101–150: more positive than negative, 151–200 excellent. DREEM has also five subscales, each of which consists of a set of items: The Students’ Perceptions of Learning (SPL) subscale measures what students think of the content of the curriculum of the educational program. It consists of 12 items and has a maximum score of 48. The Students’ Perceptions of Teachers (SPT) measures the appeal of the instructors to the students. It has 11 items and a maximum score of 44. The Students’ Academic Self-Perceptions (SASP) subscale measures if the students think that the course will be helpful in their future progress and career. It has 8 items and a maximum score of 32. The Students’ Perceptions of Atmosphere (SPA) measures the general atmosphere of the seminar and whether it was friendly and motivating or not. It consists of 12 items and has a maximum score of 48. The Students’ Social Self-Perceptions (SSSP) evaluates how students see themselves regarding their social environment and their relationship with the other participants. It consists of 7 items and has a maximum score is 28 [18, 28, 32].

After completion of the course, we asked all 303 participants to answer the DREEM questionnaire online. We also asked two extra questions (Q1. ‘Do you think that the course will prove itself beneficial to your clinical skills?’ and Q2. ‘Would you suggest the course to another student?’) with 5‑point Likert scale scores, from 0 (strongly disagree) to 4 (strongly agree). We performed a descriptive analysis of the data (DREEM, Q1, Q2).

We used SPSS v. 20 (IBM Corp., Armonk, NY, USA) in order to analyze the date and perform the appropriate tests.

## Results

Out of the 303 participants, 281 answered the questionnaire (response rate: 92.7%); 109 were males (38.8%) and 172 females (61.2%). Of the participants, 261 (92.9%) were medical students of Democritus University of Thrace, while only 20 (7.1%) were members of other medical schools in Greece. The majority of the students who attended this seminar were in the middle of their undergraduate medical training (4th and 5th year students). More specifically, 74 (26.3%) were 4th year medical students, 67 (23.8%) were in the 5th year, 46 (16.4%) in the 3rd, 36 (12.8%) in the 2nd, 26 (9.3%) in the 6th (final year), 24 (8.5%) in the 1st and only 8 (2.8%) were over the 6th year.

The overall DREEM score was 140.32 (±23.39), which is interpreted as ‘More positive than negative’ (category limits: 101–150). The subscale scores were 33.11 (±6.55) for SPL, 31.68 (±6.48) for SPT, 21.32 (±4.30) for SASP, 34.97 (±6.43) for SPA and 19.23 (±3.64) for SSSP. The interpretation of each subscale score, along with the relevant category limits are reported in Table 2. The mean scores of the extra Q1 and Q2 questions were 3.07 (±0.78) and 3.65 (±0.61) respectively.

**Table 2:** Results of the DREEM inventory

(Subscale) Question	Mean score (SD)	Score category (category limits)
Students’ perceptions of learning (SPL)	33.11 (6.55)	‘A more positive perception’ (25–36)
Students’ perceptions of teachers (SPT)	31.68 (6.48)	‘Moving in the right direction’ (23–33)
Students’ academic self-perceptions (SASP)	21.32 (4.3)	‘Feeling more on the positive side’ (17–24)
Students’ perceptions of atmosphere (SPA)	34.97 (6.43)	‘A more positive atmosphere’ (25–36)
Students’ social self-perceptions (SSSP)	19.23 (3.64)	‘Not too bad’ (15–21)
Overall score	140.32 (23.39)	‘More positive than negative’ (101–150)
Q1. Do you think that the course will prove itself beneficial to your clinical skills?	3.07 (0.78)	–
Q2. Would you suggest the course to another student?	3.65 (0.61)	–

*Q1* Extra Question 1, *Q2* Extra Question 2

The limits of Q1 and Q2 scores were 0 (for minimum) and 4 (for maximum). The maximum score for SPL, SPT, SASP, SPA, SSSP and overall is 48, 44, 32, 48, 28 and 200, respectively

## Discussion

This study concludes that medical students have a generally positive perception of the learning environment provided with the education methods employed in the Emergency Cases School. In our course, we tried to apply these methods and achieve an enhanced appeal to the audience. We used the widely used DREEM inventory for this purpose and received encouraging results. The scores indicate the sense of satisfaction of the students with this theatre-play seminar. This is in agreement with other examples of short, brief seminars which have been evaluated with the DREEM score and exhibit scores in the second highest score categories [29]. Students are equally satisfied with all the five sectors measured by the DREEM score and they find themselves attracted by the simulated clinical cases and their ability to absorb enhanced knowledge. Therein lies the greatest benefit of the theatre educational models compared with the traditional lecturing strategies. Although both traditional lecturing and theatre teaching target a large audience, the latter includes simulation techniques that seem very attractive to students.

The high ratings of the seminar could also have additional explanations. Harsh economic circumstances in Greece do not allow public institutions to provide abundant equipment and materials for educational purposes. The Greek students rarely have the chance to interact and even come in close contact with the tools, equipment and settings of a real ER environment. It is plausible that the seminar is so popular with Greek students, because it also lets them watch how these settings are practically used in a simulated environment, and offers the time to understand the diagnostic and therapeutic actions taken. These actions are later explained by the instructor. So, the learner not only feels the realism of the simulation settings, but also has the chance to step back, analyze and understand all the actions. Moreover, the quality standards according to which we selected the faculty members may have contributed to the educational efficacy of the instructors which led to these high ratings in the DREEM scores.

The distribution of the participants across different years of study shows a trend to the younger side, and especially to the 4th and 5th years. This could possibly be explained by the fact that the 6th year students have a relevantly heavy schedule which did not offer many of them the time needed to participate. Additionally, the younger students may find the novel clinical experience of the simulated ER environment more fascinating than do the older and more experienced students, and thus are more keen on participating. Moreover, the vast majority of the students came from Democritus University of Thrace, and only a small percentage of students came from other schools. The long distances of the other institutions may be the reason for this difference.

The cost of such a seminar’s set up may be higher than the cost of the traditional lecturing model, but it remains clearly lower than the cost of other widely used simulation models, which include multiple stations with fewer participants in each one [33, 34]. Our seminar trained over 300 students and cost approximately €550, from which €430 was spent on the lunch break and the material distributed to the students, and thus these costs could be decreased. The equipment required was kindly donated by our university and thus, the basic cost of the seminar was extremely low. However, even if we had to pay for the equipment and the tools used, the cost would still be relevantly lower than in other seminars with multiple simulation stations, as the latter setting would require multiple sets of tools and pieces of equipment (from needles and oxygen masks to electrocardiographs and beds) for the stations which would run simultaneously. The relatively low cost makes this role-playing setting more economically feasible and accessible to more attendants compared with other multiple-stations settings. However, it preserves the realism and the pressure of the ER, in contrary to the traditional lecturing model, and thus it leads to high ratings when evaluated for the educational environment it creates. Thus, theatre educational structures, such as Emergency Cases School, could play the role of the ‘golden mean’ between effectively training a large number of students and maintaining the cost low. This is especially important when it comes to organizations and institutions with limited financial resources, such as the Greek medical education system, while at the same time increasing the efficiency and appeal of the medical school curriculum. We have to remember that times of significant economic challenges, such as the current one in Greece, also represent opportunities to achieve constructive changes.

One of the limitations of the study is that the DREEM questionnaire only addresses the opinions of the attendants of the course, while its actual impact on the students’ performance and skills has not been measured in the long term. The financial resources needed for the different ways of teaching could also be measured to make a more accurate and extended comparison.
